# Splitting ore from X-ray image based on improved robust concave-point algorithm

**DOI:** 10.7717/peerj-cs.1263

**Published:** 2023-02-23

**Authors:** Lanhao Wang, Hongdong Hu, Zhaopeng Li, Wei Dai

**Affiliations:** 1 National Engineering Research Center of Coal Preparation and Purification, China University of Mining Technology, Xuzhou, China; 2 School of Information and Control Engineering, China University of Mining Technology, Xuzhou, China; 3 LONGi Magnet Co., LTD., Shenyang, China

**Keywords:** Ore separation, Image segmentation, Concave point matching

## Abstract

Image segmentation is a key part of ore separation process based on X-ray images, and its segmentation result directly affects the accuracy of ore classification. In the field of ore production, the conventional segmentation method is difficult to meet the requirements of real-time, robustness and accuracy during ore segmentation process. In order to solve the above problems, this article proposes an ore segmentation method dealing with pseudo-dual-energy X-ray image which is composed of contour extraction module, concave point detection module and concave point matching module. In the contour extraction module, the image is firstly cut into two parts with high and low energy, then the adaptive threshold is used to obtain the ore binary image. After filtering and morphological operation, the image contour is obtained from the binary image. Concave point detection module uses vector to detect concave points on contour. As the main contribution of this article, the concave point matching module can remove the influence of boundary interference concave points by drawing the auxiliary line and judging the relative position of auxiliary line and ore contour. With the matching concave points connected, the whole ore segmentation is completed. In order to verify the effectiveness of this method, a comparative experiment was conducted between the proposed method and conventional segmentation method using X-ray images of antimony ore as data samples. The result of industrial experiment shows that the proposed intelligent segmentation method can remove the interference of pseudo concave points on the contour, achieve accuracy segmentation result, and satisfy the requirements of processing X-ray image of ore.

## Introduction

In the process of ore production, separation is an indispensable procedure, the effect of ore separation directly determines whether the raw ore can be fully utilized. At present, the main beneficiation methods are hand separation, gravity separation, floating separation, and so on (Qin et al., 2017). Among them, hand selection is based on the difference in color, luster and shape between target minerals and gangue in raw ore. Although laborious, this method can often obtain a higher grade concentrate. Gravity separation makes use of the density difference of ore and gangue. Floating separation is the main method for mineral extraction, but it is not suitable for all minerals. Taking antimony ore as an example, while antimony sulfide is a floating minera, antimony oxide belongs to refractory ore. Above methods have very significant problems, such as low identification accuracy, requiring of large space and higher investment costs, *etc*.

With the development of computer science and image processing technology, machine vision has been applied in the field of mineral separation (Jung & Choi, 2021) in recent years. Separation method based on dual-energy X-ray has attracted more and more attention. Scholars have carried out a lot of research on mineral separation. While most of the research focuses on how to conduct the separation according to the physical characteristics of ore itself (Von Ketelhodt & Bergmann, 2010), there are few researches on the separation of adherent ores.

The traditional image segmentation process mainly uses the methods based on threshold, edge, region and clustering. The essence of image segmentation method based on threshold (Otsu, 1979) is to classify image gray histogram by setting different gray threshold values (Huang, Zheng & Liang, 2020). Edge detection (Rosenfeld, 1981) consists of serial edge detection and parallel edge detection (Khan, Bhuiyan & Adhami, 2011). The serial edge detection method first detects the starting point of the edge, from which the adjacent edge points are searched and connected by the similarity criterion to complete the image edge detection; The parallel edge detection method is segmented by using the spatial calculus algorithm and convolving its template with the image in parallel. In practice, the parallel edge detection method can complete the segmentation by convolution directly with differential operators such as Robers (Rosenfeld, 1981), Sober (Gao et al., 2010) and Canny (Er-sen et al., 2009). The region based image segmentation method uses the spatial information of the image for classification, and there are many methods (Pham, Xu & Prince, 2000), among which the region growing algorithms, spliting and merging algorithm (Tremeau & Borel, 1997) and watershed algorithm (Chandra, Supraja & Bhavana, 2017) are the most commonly used methods. The region growing algorithms collects pixels with similar properties to form independent regions to get segmentation results. The essence of the spliting and merging algorithm is to get each sub-region of the image by constantly splitting and merging. The watershed algorithm (Liu, 2019; Chien, Huang & Chen, 2003) treats the image it operates as a topographic map, in which the brightness value of each pixel represents its height. The image segmentation method based on clustering (Huang, Zheng & Liang, 2020, Rosenfeld, 1981) gathers pixels with similar features into the same area, iterates and converges the clustering results repeatedly, and finally divides all pixels into several different categories to get the segmentation results.

With the development of deep learning, convolutional neural network has been introduced into the field of image segmentation as an important means of image processing. It can make full use of the semantic information of the image to realize the segmentation of the image. A series of image semantic segmentation methods based on deep learning, such as FCN (Long, Shelhamer & Darrell, 2015), PSPNet (Zhao et al., 2017), DeepLab (Chen et al., 2016, 2017) and Mask R-CNN (He et al., 2017), have been proposed. However, although the deep learning method has strong adaptability, it still has some shortcomings, such as requiring a large number of datasets for training and hard to obtain real-time segmentation results.

For specific segmentation scenarios, some of the algorithms can achieve good segmentation result. However, the result of ore adhesion is varied, thus the method mentioned cannot be directly applied to ore segmentation. The method based on concave point detection is a very effective method to segment circular adhesion objects. It can be observed that there must be multiple concave points existing in the outline when circular objects adhere to each other, and we can use this priori knowledge to segment such adhesive objects. For example, Yao et al. (2017) uses the concave point detection algorithm to segment rice, and Song, Zhao & Liu (2014) proposes a method combining the concave point detection and watershed algorithm to segment the adhesion cells. The segmentation method based on concave point detection is not only fast enough to meet the needs of tasks requiring high real-time performance, but also can achieve good results for objects with smooth surface such as cells. However, although the ore is also a kind of circular object, its physical characteristics determine that there are many small interference concave points on its surface. If the conventional concave point detection method is used directly, these interference concave points will not be filtered out, which will inevitably lead to a large amount of over-segmentation phenomena.

To solve the above problems, inspired by the traditional concave point detection algorithm, a new concave point matching algorithm is proposed in this article to filter the small concave points on the edge caused by the ore’s own characteristics, and the proposed method can greatly improve the accuracy of concave point matching. The proposed algorithm includes three parts: contour extraction module using adaptive threshold segmentation and image binarization and Suzuki’s contour extration algorithm (Suzuki & Abe, 1985), concave point detection module based on vector angle and concave point matching module based on concave point auxiliary line. In order to validate the proposed method, industrial experiment have been carried out on the antimony ore dataset and compared with other methods. The experiment show that the proposed concave matching method based on the auxiliary line of the concave point can well remove the small interference concave points, and meet the requirements of ore X-ray image segmentation.

## Image segmentation of ore

### Industrial background

**Figure 1:**
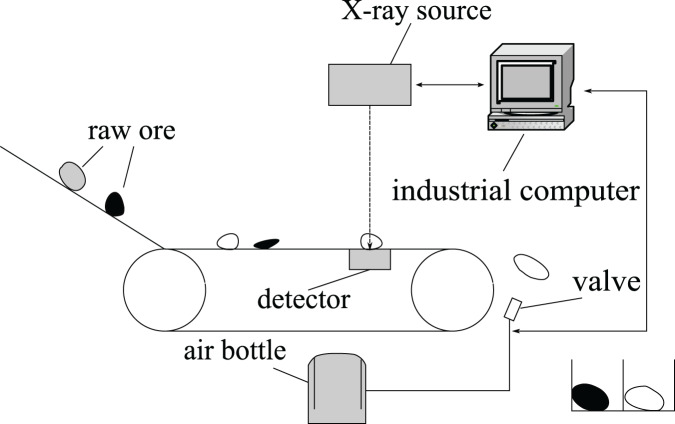
Ore sorting device.

The typical structure of ore sorting device based on X-ray is shown in Fig. 1. The sorting process is as follows: first, raw ore is crushed into small stones of uniform size, which are sent to the pseudo-dual-energy X-ray identification equipment through the belt conveyor. Then, the industrial computer needs to determine the grade of ore from the high and low energy images obtained from the X-ray device and pass the coordinate of ore to the valve controller, which uses the coordinates to adjust the direction of the air flow to blow the ore into the correct separator box. In this process, the image segmentation algorithm must be able to get the correct position of the ore from the X-ray image, otherwise the subsequent valve controller will not work correctly.

**Figure 2:**
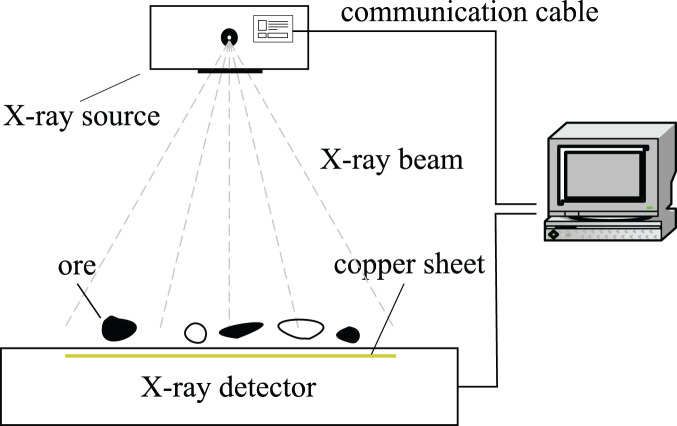
X-ray processing subsystem.

The pseudo-dual-energy X-ray processing subsystem used in the system is shown in Fig. 2. It contains an X-ray source and a pseudo-dual-energy X-ray detector. The X-ray source emits X-ray beam with a continuous spectrum, and the dual-energy detector is a two-layer structure, the upper layer collects low-energy signals, the middle layer uses a copper sheet to filter out the low-energy part of the ray, and the lower layer collects high-energy signals. The pseudo-dual-energy X-ray system is widely used in industrial inspection due to its simple structure, high precision and cost performance.

**Figure 3:**
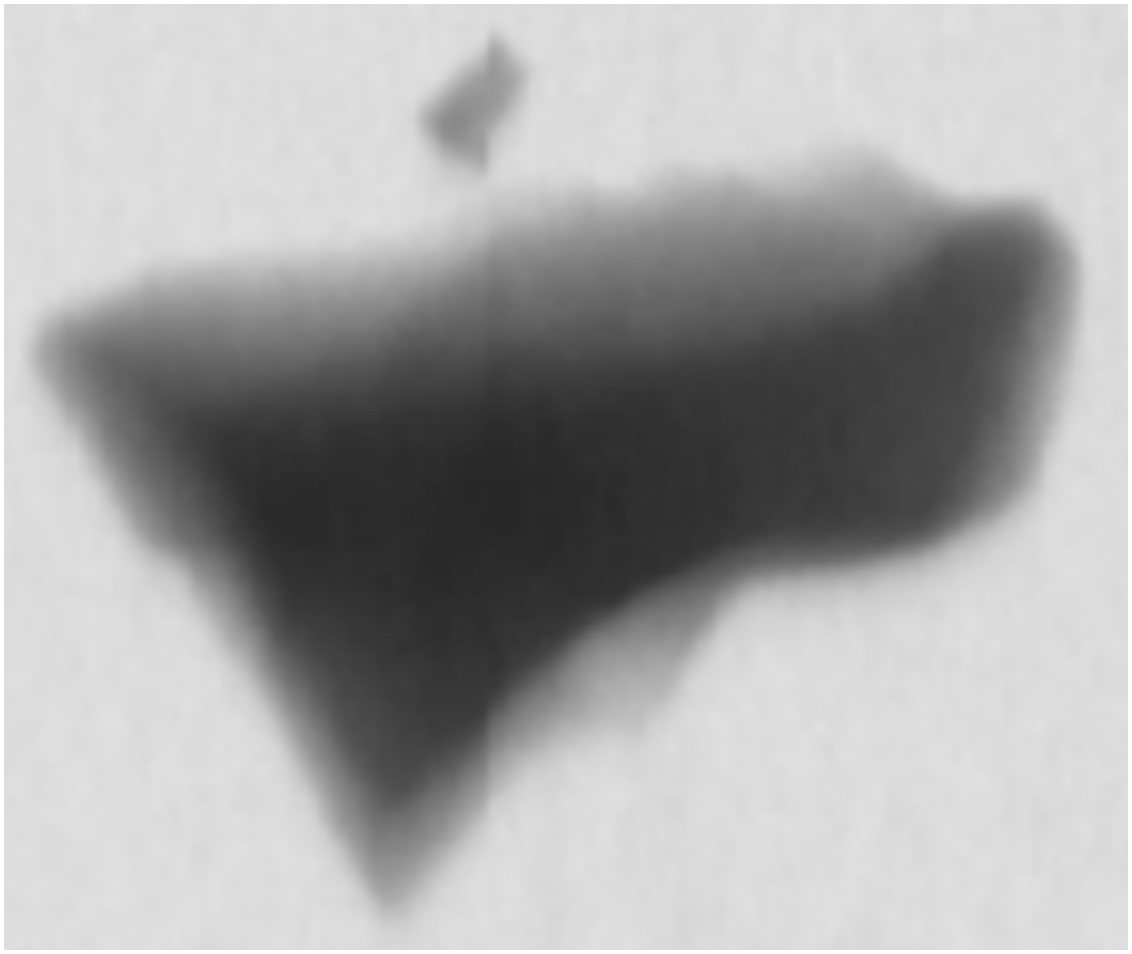
Gray image obtained from X-ray detector.

At present, the mainstream sorting method based on pseudo-dual-energy X-ray relies on the fact that different grades of ore have different physical characteristics and thus have different absorption ability for X-ray, so that the gray level of the picture obtained from the X-ray detector is different (one example is shown in Fig. 3). When the ore is separated from the original image, through these gray features, combined with the classification algorithm, different grades of ore and gangue can be separated.

### The difficulty of ore image segmentation based on x-ray

#### Real time performance

Industrial computers must segment, classify ore from image and send ore locations and categories to subsequent controller in limited time. If the algorithm takes too long time, the antimony will not be blown into the corresponding box by the air valve in time, which will lead to the failure of sorting.

#### Accuracy

The classification algorithm uses each pixel of the segmented ore to classify the mineral. If over-segmentation occurs, the number of pixels available for ore classification will be reduced, resulting in errors in classification results. Accordingly, if undersegmentation occurs, the classification results will be inaccurate because the samples to be tested are mixed with different kinds of ores.

#### Robustness

In the actual production process, there will be a lot of small stones and other debris on the belt, and eventually these debris will appear on the X-ray gray image in the form of noise. If these noises are not processed, over-segmentation will occur and the accuracy of recognition will be reduced.

## Robust ore image segmentation algorithm

The ore image segmentation algorithm proposed in this article is implemented on the system shown in Fig. 1. It consists of three modules: contour extraction module, concave point detection module and concave point matching module. The contour extraction module consists of image cutting, image binarization, noise processing and contour extraction. The concave point detection module determines whether the point on the contour is concave by calculating the angle between three consecutive points. As the main contribution of this article, the concave point matching module filters out the interference concave points on the boundary by drawing parallel lines of connecting lines of concave points and judging the relative position of parallel lines and ore contours, so as to reduce the probability of over-segmentation.

### Image cutting

**Figure 4:**
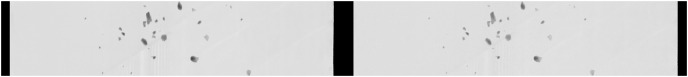
Original image.

**Figure 5:**
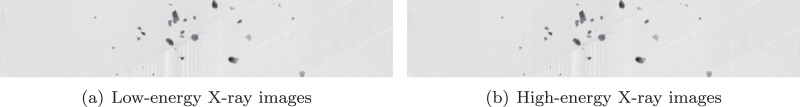
(A–B) Two parts of X-ray image.

As shown in Fig. 4, the output image of the X-ray processing subsystem consists of two parts: high-energy part and low-energy part, so it needs to be cut apart through the slicing operation of the matrix. The image obtained after cutting is shown in Fig. 5.

### Image binarization

In order to find the ore contour using Suzuki’s method (Suzuki & Abe, 1985), the image obtained in the Image cutting section needs to be binarized. Fixed threshold segmentation, Otsu threshold segmentation, and adaptive threshold segmentation are the most commonly used methods. It is found in practice that for the samples obtained from actual production process, fixed threshold and Otsu threshold segmentation, as a global threshold segmentation method, cannot effectively binarize ore image under the condition of dark background, much noise and interference caused by stone powder, while as a method using local threshold, adaptive threshold segmentation can better adapt to complex situations in different scenes. Therefore, this article adopts adaptive threshold segmentation algorithm to segment non-adhesive images, and carries out preliminary segmentation of adhesive images.

**Figure 6:**
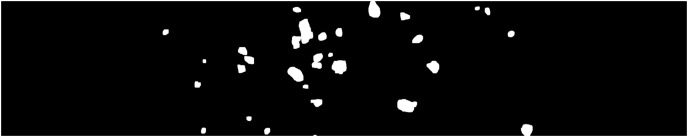
The binary image obtained by adaptive threshold segmentation.

By selecting the low energy image or high energy image in Fig. 5, the binary image can be obtained after being processed with the adaptive threshold segmentation algorithm, and the result is shown in Fig. 6.

### Noise filtering

In the actual production process, the binary image of ore has a lot of noise. If the noise is not filtered, the program will treat the noise as tiny ores, which not only increases the computing load of the computer, but also interferes with the process of recognizing normal ores.

**Figure 7:**
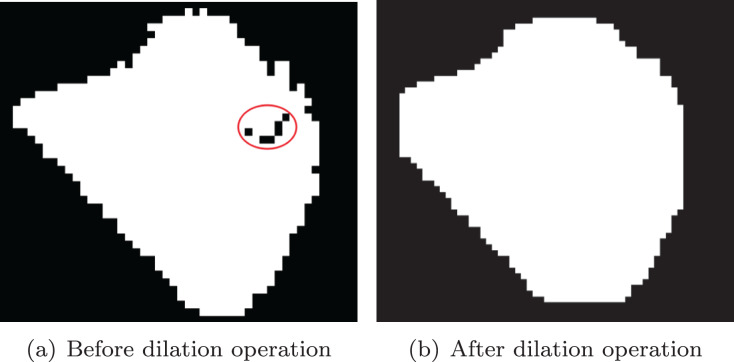
Dilation operation. (A) Before dilation operation; (B) after dilation operation.

**Figure 8:**
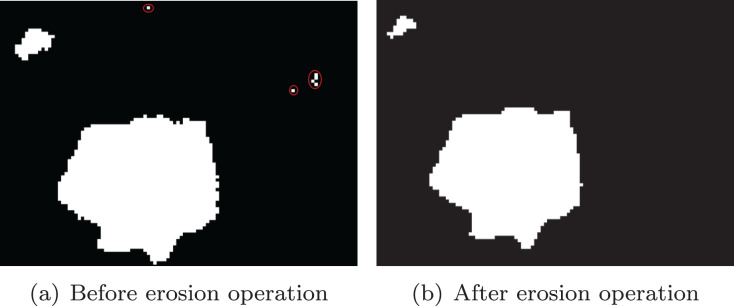
Erosion operation. (A) Before erosion operation; (B) after erosion operation.

We use morphological operation (Comer & Delp, 1999) to remove noise from the image. Firstly, the noise inside the stones were removed by dilation operation, as shown in Fig. 7. Similarly, in order to remove noise outside the ore, erosion operation is used, and the effect is shown in Fig. 8.

### Contour extraction

**Figure 9:**
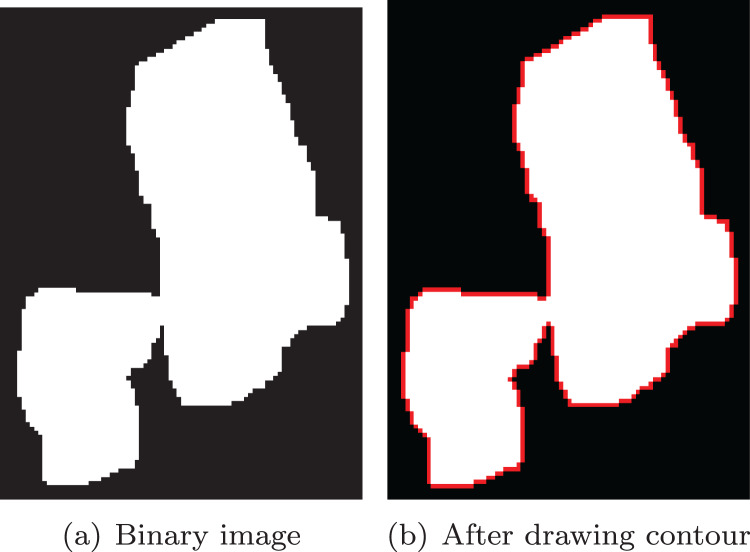
Extract the contour from the binary image. (A) Binary image; (B) after drawing contour.

In this article, the method proposed by Suzuki (Ren, Zhang & Zhang, 2019, Suzuki & Abe, 1985) is used to extract contour from binary image, and the extracted contour is shown in Fig. 9.

### Concave point detection

It can be observed that the concave point is a class of points with the maximum local curvature on the contour formed by two or more circular objects stacked. For a single smooth elliptic object, the contour curve will not have a large curvature mutation, nor will it form a concave region caused by overlapping of different objects. On the contrary, there must be points whose curvature change suddenly on the contour of the cohesive ores, which is the basis for determining concave points.

**Figure 10:**
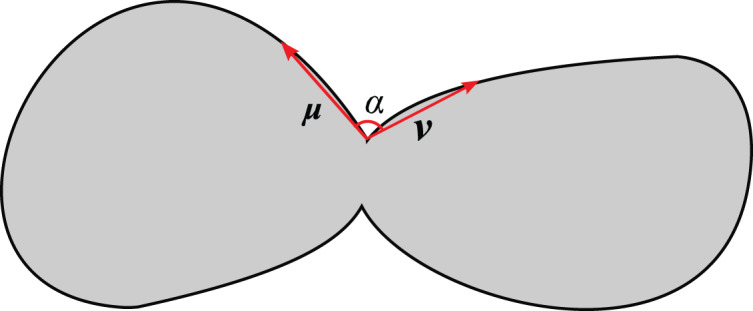
Concave point detection.

As shown in Fig. 10, two vectors named ${{u}}$ and ${{v}}$ are formed by three consecutive points on the contour (the length of the ${{u}}$ and ${{v}}$ are greater than the hyperparameter d), then the angle between the two vectors is calculated to obtain the concavity corresponding to the point.

For points on the contour $[{p_1},{p_2},...,{p_k},...,{p_n}]$, in order to find the concavity corresponding to point ${p_k}$ on the contour, by using Eq. (1), $m = i$ and $m = j$ can be solved separately (in Eq. (1), $i \gt k,j \lt k$, and in this article, d is set to 6).

(1) $$\eqalign {&{\mathop \arg \min \limits_{\hskip -15pt m}} |k - m| \cr& {{s.t.}\ {|{p_k} - {p_m}| \gt d}}}$$

By substituting the values of $i$ and $j$ into Eqs. (2) and (3), we can derive the values of the vectors ${{u}}$ and ${{v}}$, and their corresponding coordinates ${u_x}$, ${u_y}$, ${v_x}$ and ${v_y}$.

(2) $${{u}} = ({u_x},{u_y}) = {p_i} - {p_k}$$

(3) $${{v}} = ({v_x},{v_y}) = {p_j} - {p_k}$$

To calculate the angle between the vectors ${{u}}$ and ${{v}}$, the function $\theta (x,y)$ defined in Eq. (4) is used to calculate the angle between the x-axis and the vector ${{u}}$, and the same is true for ${{v}}$, so that we can obtain the values of $\theta ({u_x},{u_y})$ and $\theta ({v_x},{v_y})$. Let ${\theta _1}$ be the difference between $\theta ({u_x},{u_y})$ and $\theta ({v_x},{v_y})$. Note that $\theta (x,y)$ in Eq. (4) is 0 in the positive direction of the x-axis, and gradually increases in the counterclockwise direction, with a max value of $2\pi$.

(4) $$\theta (x,y) = \left\{ {\matrix{ {arctan({y \over x})} \hfill & {x \gt 0,y \ge 0} \hfill \cr  {arctan({y \over x}) + \pi } \hfill & {x \lt 0} \hfill \cr  {arctan({y \over x}) + 2\pi } \hfill & {y \lt 0,x \gt 0} \hfill \cr  {{\pi \over 2}} \hfill & {x = 0,y \gt 0} \hfill \cr  {{{3\pi } \over 2}} \hfill & {x = 0,y \lt 0} \hfill \cr  } } \right.$$

(5) $${\theta _1} = \theta ({u_x},{u_y}) - \theta ({v_x},{v_y})$$

As ${\theta _1} \in [ - 2\pi ,2\pi ]$, the result obtained in the previous step need to be processed by using Eq. (6) to obtain the angle $\alpha$.

(6) $$\alpha = \left\{ {\matrix{ {{\theta _1} + 2\pi } \hfill & {{\theta _1} \in ( - 2\pi , - \pi )} \hfill \cr  {{\theta _1}} \hfill & {{\theta _1} \in ( - \pi ,\pi )} \hfill \cr  {{\theta _1} - 2\pi } \hfill & {{\theta _1} \in (\pi ,2\pi )} \hfill \cr  } } \right.$$

**Figure 11:**
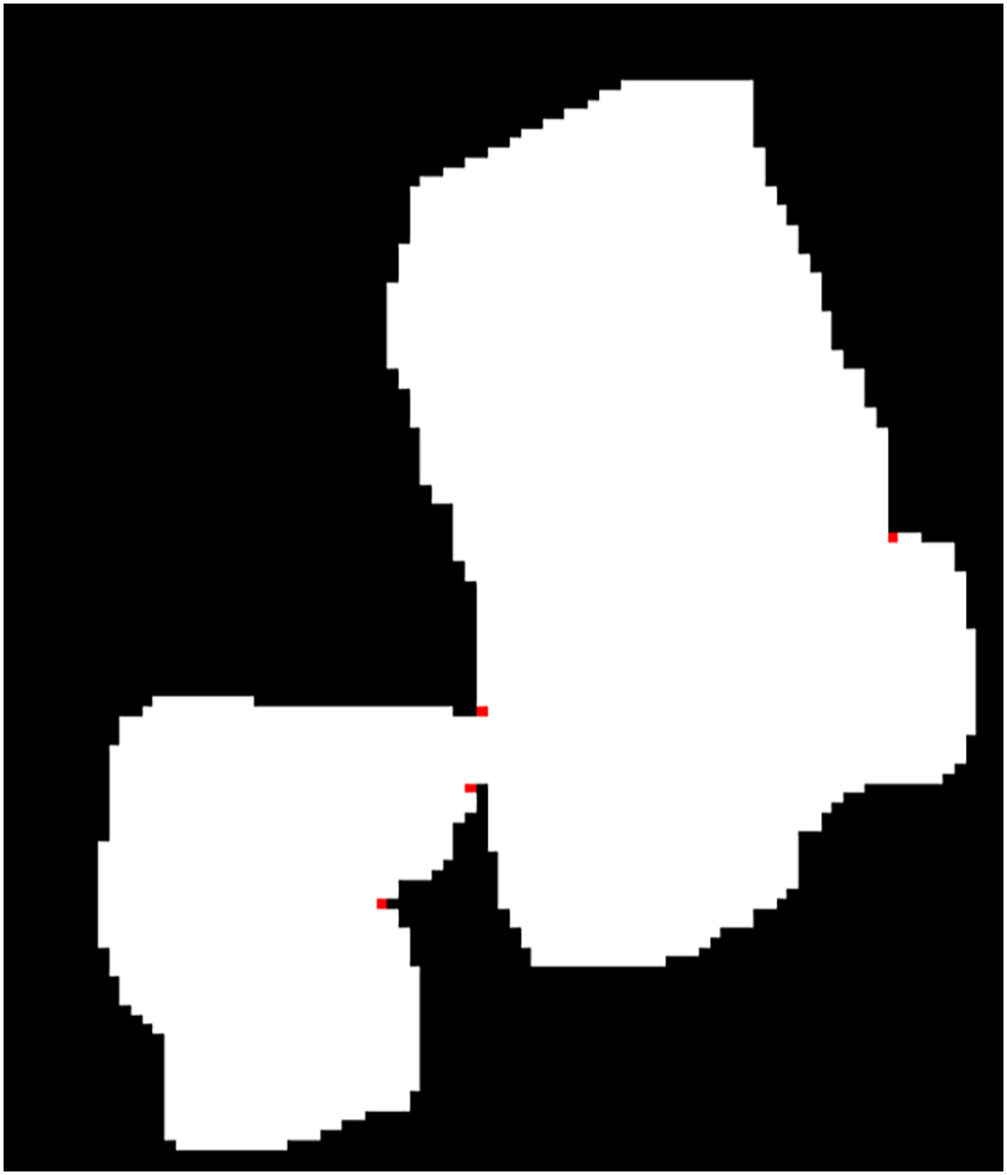
Concave points detected on the contour.

We can obviously judge the convexity and angle of the local contour from the value of $\alpha$, that is, if $\alpha \in [0,\pi ]$, it is concave, otherwise it is convex. In practical use, in order to reduce interference, relatively flat local contours will be excluded, that is, point on the contour can be regarded as a concave point only if $\alpha \in [0,{\theta _t}]$, where ${\theta _t} \lt \pi$. After repeated experiments, it is found that selecting ${\theta _t} = {{3\pi } \over 4}$ as threshold in this article can achieve better detection effect. By using the concave detection algorithm described above, the concave points can be detected in the extracted contour (b) in Fig. 9, and these concave points are annotated in red in Fig. 11 (the contour drawn in the Contour extraction section is hidden to highlight the concave points).

### Concave point matching

**Figure 12:**
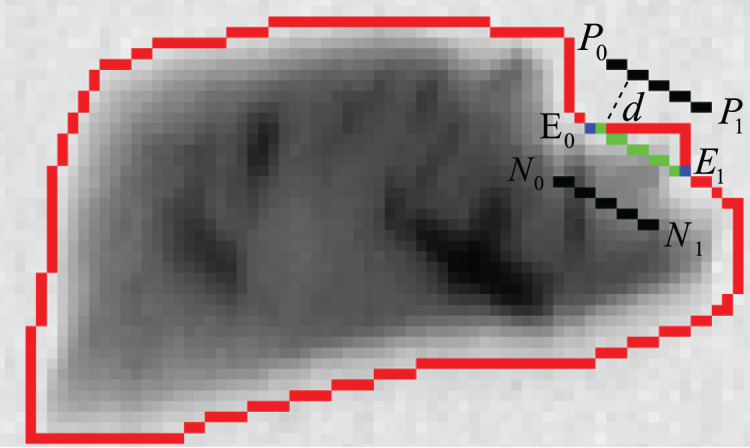
Three-line method.

The physical characteristics of the ore often lead to the existence of a large number of concave points on its edge in the absence of adhesion with other ores. For example, because some antimonite exists in the form of crystal, many concave points will be detected when the concave detection algorithm is performed. For this kind of ore, it will lead to over-segmentation if the concave line is used as the dividing line directly. This problem can be solved perfectly by using three-line method proposed in this article. The schematic diagram of this method is shown in Fig. 12.

As can be seen from the Fig. 12, due to the characteristics of the ore itself, concave points can be detected even from the outline of a single ore. Obviously, in such case, the connecting line of the concave points cannot be directly used as the dividing line (marked in green). In order to eliminate the false segmentation caused by such points, auxiliary lines are drawn in the figure (marked in black). The connecting line of its concave points can be used as a dividing line if the auxiliary lines are all inside the contour. The detailed steps of the method are as follows:

#### Finding the auxiliary line

As shown in the Fig. 12, the auxiliary line is actually the parallel line of the connecting line of the two concave points, and it has the same length as the connecting line of the concave points. Therefore, the auxiliary line can be obtained by combining the slope $k$ of the connecting line between the concave points and the given distance d.

Let ${E_0} = ({r_0},{c_0})$ and ${E_1} = ({r_1},{c_1})$ be the endpoints of the connecting line. First, calculate its slope $k$ by using Eq. (7).

(7) $$k = {{{c_1} - {c_0}} \over {{r_1} - {r_0}}}$$

The distance difference ( ${\Delta _x}$ and ${\Delta _y}$) between the concave point and the end point of the auxiliary line is obtained by using slope $k$ and distance $d$.

(8) $${\Delta _x} = {{k \cdot d} \over {\sqrt {1 + {k^2}} }}$$

(9) $${\Delta _y} = {{ - {\Delta _x}} \over k}$$

The endpoints of the concave points $({r_0},{c_0})$ and $({r_1},{c_1})$ are translated by distance ${\Delta _x}$ and ${\Delta _y}$ in the horizontal and vertical directions to obtain the endpoints of the auxiliary line: ${P_0}$, ${P_1}$, ${N_0}$ and ${N_1}$.

(10) $${P_0} = ({r_0} + {\Delta _x},{c_0} + {\Delta _y})$$

(11) $${P_1} = ({r_1} + {\Delta _x},{c_1} + {\Delta _y})$$

(12) $${N_0} = ({r_0} - {\Delta _x},{c_0} - {\Delta _y})$$

(13) $${N_1} = ({r_1} - {\Delta _x},{c_1} - {\Delta _y})$$

Wire ${P_0}$ and ${P_1}$ to get the auxiliary line ${L_P}$. Similarly, the auxiliary line ${L_N}$ can be obtained by connecting ${N_0}$ and ${N_1}$.

#### Judging the position relation between auxiliary line and contour

**Figure 13:**
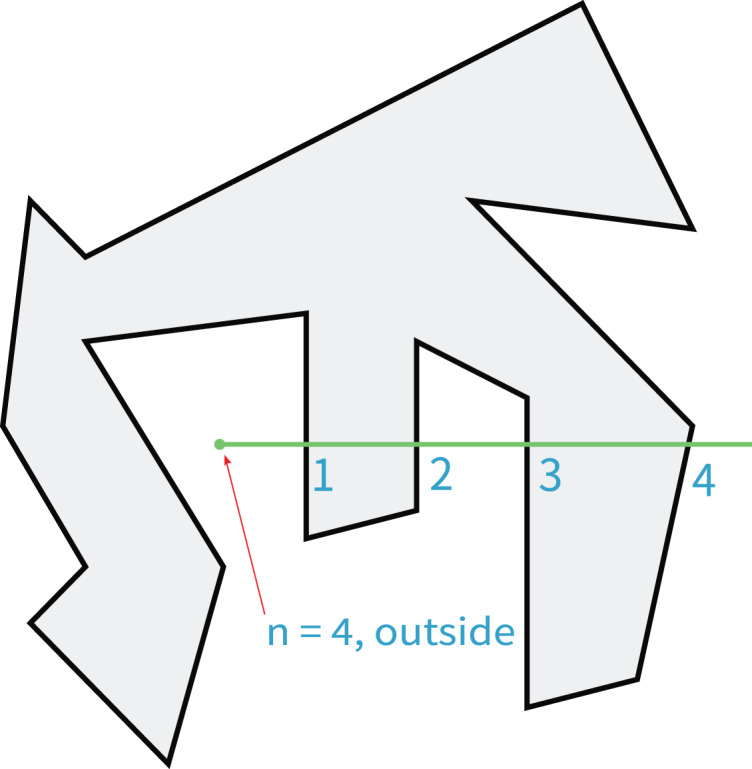
Find the position relation between any point and the contour.

In order to judge the position relationship between the auxiliary line and the contour, it is necessary to judge the position relationship between each point of the auxiliary line and the contour. In this article, the PNPoly algorithm (Haines, 1994; Zhang & Zhou, 2022) proposed by W. Randolph Franklin is used to solve the position relationship between the point and the contour (the point to be measured can be on the contour, inside the contour or outside the contour). The idea of the algorithm is shown in Fig. 13.

As can be seen from the figure, the position relationship between the point and the contour can be judged by drawing the horizontal line and finding the number of times that the line crosses the contour.

#### Finding the true dividing line

As shown in Algorithm 1, by repeatedly applying the method described in the Concave point detection section and the Concave point matching section for each candidate concave point splitting line, the true splitting line of the adhered ore can be found.

**Algorithm 1:** Finding the true dividing line

**Input:** C: Concave point set
**Output:** *L*_*list*_: Candidate dividing lines set
1: For concave point set $C = \{ {p_1},{p_2},...,{p_n}\}$, after performing the permutation and combination operation, we get $\{ {p_1},{p_2}\}$, $\{ {p_1},{p_3}\}$, $\{ {p_i},{p_j}\}$,…, $\{ {p_{n - 1}},{p_n}\}$
2: Calculate the distance d between its elements: $D = \{ {d_{1,2}},{d_{1,3}},{d_{i,j}},{d_{n - 1,n}}\}$, ${d_{i,j}} = |{p_i}{p_j}|$
3: Find the minimum value ${d_{k,q}}$ in set D
4: Add the connecting line *L* between *p*_*k*_ and *p*_*q*_ into *L*_*list*_
5: Remove ${p_{k,t}}$ and ${p_{t,q}}$ from set *D*,where $t = 1,2,...,n$
6: Repeat steps 3–5 until the number of elements in C is less than 2
7: **for all** line *L* **in** the *L*_*list*_
8: Let distance be *d*, draw auxiliary line *L*_*siblings*_
9: **if** *L*_*siblings*_ are all on the contour **then**
10: Append L into *L*_*list*_
11: **end if**
12: **end for**
13: **return** *L*_*list*_

### Complete segmentation process

**Figure 14:**
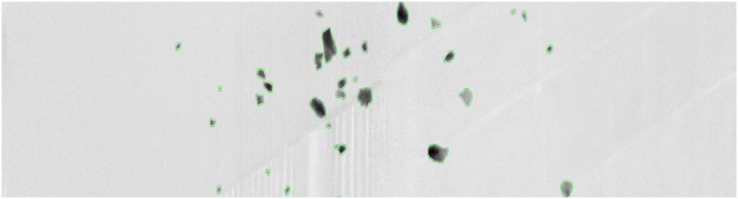
Segmentation example.

The overall segmentation process is shown in the Algorithm 2, one of the segmentation examples is shown in Fig. 14.

**Algorithm 2:** Overall segmentation process

**Input:** Pseudo-dual-energy X-ray grayscale image
**Output:** Contour of ore
1: Image cutting
2: Image binarization
3: Noise filtering
4: Contour extraction
5: Concave point detection
6: Using concave point mathing algorithm proposed in this article to
get the splitting line if the number of concave points is bigger than 2
7: The dividing line is combined with the contour in step 4, and the
result is used as the new segmentation contour
8: **return** The segmented contour

## Experiments

**Figure 15:**
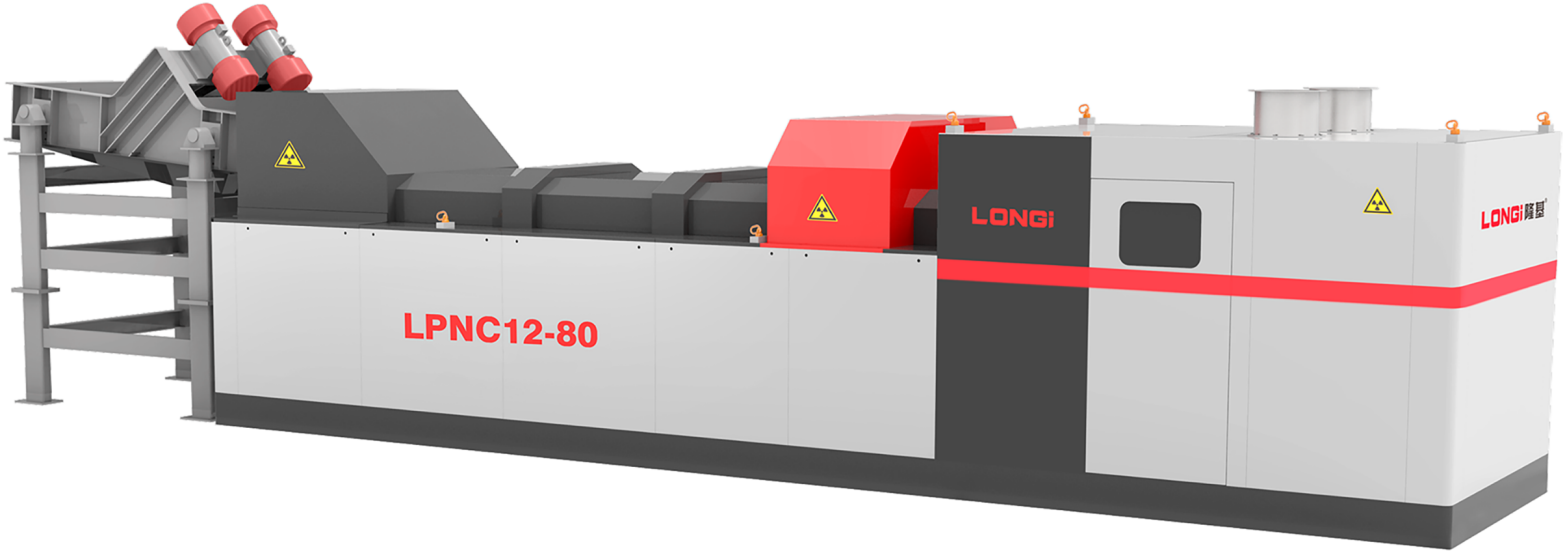
LNPC12-80.

LNPC12-80, a pseudo-dual-energy X-ray sorting device produced by Longi company, was selected for the experiment, the equipment shown in Fig. 15.

In order to verify the performance of the proposed algorithm, the antimony ore dataset made by ourselves is selected for the experiment. The relevant parameters of the sample are shown in Table 1.

**Table 1:** Sample distribution

Types of antimony ore	Weight	Grade
High grade ore	1.91 kg	$26.83\%$
Medium grade ore 1	1.7 kg	$4.74\%$
Medium grade ore 2	4.8 kg	$0.556\%$
Tailings	17.9 kg	$0.024\%$

In this experiment, the memory of the host used in the experiment is 16G, the CPU model is ${\rm{28375CX2}}$, and the windows 10 operating system is used. C++ and Opencv library are used to implement the proposed algorithm, and 134 single-channel $512 \times 5,632$ gray scale images are tested.

### Analysis of results

**Figure 16:**
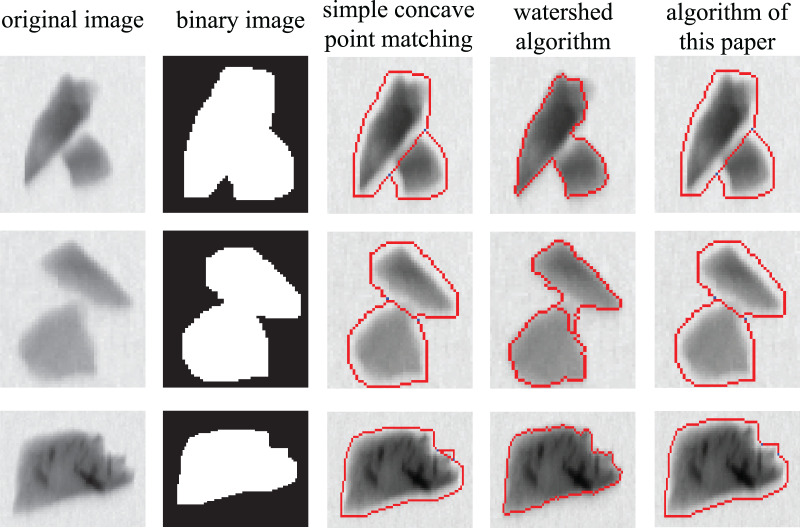
Comparison of different algorithms.

In order to better demonstrate the superiority of the algorithm in this article, the algorithm proposed in this article is compared with simple concave point matching algorithm and the watershed algorithm based on distance transform using exactly the same dataset. Several typical images are selected from the results of experiment to illustrate the performance of each algorithm. As shown in Fig. 16, if the overlapping area of two ores is large, they cannot be separated by the watershed algorithm (as shown in the first row). Even if the watershed algorithm can be used to divide the cohesive ore, the dividing line in the segmentation result is not very accurate (as shown in the second row). Due to the physical characteristics of the ore, even a single ore may have a large number of concave points on its contour, in this case, the use of simple concave matching will inevitably lead to over-segmentation (as shown in the third row). In the above cases, the algorithm proposed in this article can work well.

In this article, ${P_u}$ (under-segmentation rate), ${P_o}$ (over-segmentation rate) and ${P_a}$ (accuracy rate) are used as performance indicator to reflect the performance of algorithms when applied in practical industrial applications and they are defined as:

(14) $${P_u} = {{{N_u}} \over M} \times 100\%$$

(15) $${P_o} = {{{N_o}} \over M} \times 100\%$$

(16) $${P_a} = {{{N_a}} \over M} \times 100\%$$

In this formula, ${N_u}$ is the number of under-segmentation ore, ${N_o}$ is the number of over-segmentation ore, ${N_a}$ is the number of ores correctly divided and *M* is the total number of ores tested ( $M = 134$).

As can be seen from the Table 2, compared with other algorithms, the proposed algorithm can significantly reduce the probability of under-segmentation and over-segmentation and improve the accuracy of segmentation.

**Table 2:** The accuracy rate of different algorithms

Method	${P_u}\%$	${P_o}\%$	${P_r}\%$
Simple concave point matching	3.73	7.46	88.81
Watershed algorithm	5.22	2.24	92.54
Algorithm of this article	3.73	0	96.27

## Conclusion

This article presents a strategy of ore segmentation based on concave point detection. This strategy contains two main innovation. Firstly, an ore segmentation framework for pseudo-dual-energy X-ray images is proposed, which is mainly composed of contour extraction module, concave point detection module and concave point matching module. Secondly, in order to reduce the influence of the concave points caused by the physical characteristics of ore on segmentation process, this article proposes a concave point matching algorithm which uses the position relationship between the auxiliary line and the contour to judge whether the candidate segmentation scheme is available. By comparing the result of experiment, it is found that the proposed algorithm can obtain a satisfactory segmentation effect, and it can be applied to the actual industrial ore separation process, and also pave the way for the ore classification.

## Additional Information and Declarations

### Data Availability

The following information was supplied regarding data availability: The raw data and code are available at GitHub: https://github.com/GoWithWind2015/concave_paper_code. The data is also available at Zenodo: GoWithWind2015. (2023). GoWithWind2015/concave_article_code: v0.2 (v0.2). Zenodo. https://doi.org/10.5281/zenodo.7558583.

## References

[ref-1] Chandra JN, Supraja BS, Bhavana V (2017). A survey on advanced segmentation techniques in image processing applications.

[ref-2] Chen L-C, Papandreou G, Kokkinos I, Murphy K, Yuille AL (2016). Semantic image segmentation with deep convolutional nets and fully connected CRFs. ArXiv preprint.

[ref-3] Chen L-C, Papandreou G, Schroff F, Adam H (2017). Rethinking atrous convolution for semantic image segmentation. ArXiv preprint.

[ref-4] Chien S-Y, Huang Y-W, Chen L-G (2003). Predictive watershed: a fast watershed algorithm for video segmentation. IEEE Transactions on Circuits and Systems for Video Technology.

[ref-5] Comer ML, Delp EJ (1999). Morphological operations for color image processing. Journal of Electronic Imaging.

[ref-6] Er-sen L, Shu-long Z, Bao-shan Z, Yong Z, Chao-gui X, Li-hua S (2009). An adaptive edge-detection method based on the canny operator.

[ref-7] Gao W, Zhang X, Yang L, Liu H (2010). An improved Sobel edge detection.

[ref-8] Haines E (1994). Point in polygon strategies. Graphics Gems.

[ref-9] He K, Gkioxari G, Dollar P, Girshick R (2017). Mask R-CNN.

[ref-10] Huang P, Zheng Q, Liang C (2020). Review of image segmentation methods (in Chinese). Journal of Wuhan University (Science Edition).

[ref-11] Jung D, Choi Y (2021). Systematic review of machine learning applications in mining: exploration, exploitation, and reclamation. Minerals.

[ref-12] Khan JF, Bhuiyan SMA, Adhami RR (2011). Image segmentation and shape analysis for road-sign detection. IEEE Transactions on Intelligent Transportation Systems.

[ref-13] Liu Z (2019). Image segmentation of cohesive circular objects based on concave point and center of gravity detection.

[ref-14] Long J, Shelhamer E, Darrell T (2015). Fully convolutional networks for semantic segmentation.

[ref-15] Otsu N (1979). A threshold selection method from gray-level histograms. IEEE Transactions on Systems, Man, and Cybernetics.

[ref-16] Pham DL, Xu C, Prince JL (2000). A survey of current methods in medical image segmentation. Annual Review of Biomedical Engineering.

[ref-17] Qin X, Deng J, Lai H, Zhang X (2017). Beneficiation of antimony oxide ore: a review. Russian Journal of Non-Ferrous Metals.

[ref-18] Ren M, Zhang Q, Zhang J (2019). An introductory survey of probability density function control. Systems Science & Control Engineering.

[ref-19] Rosenfeld A (1981). The max roberts operator is a hueckel-type edge detector. IEEE Transactions on Pattern Analysis and Machine Intelligence.

[ref-20] Song H, Zhao Q, Liu Y (2014). Splitting touching cells based on concave-point and improved watershed algorithms. Frontiers of Computer Science.

[ref-21] Suzuki S, Abe K (1985). Topological structural analysis of digitized binary images by border following. Computer Vision, Graphics, and Image Processing.

[ref-22] Tremeau A, Borel N (1997). A region growing and merging algorithm to color segmentation. Pattern Recognition.

[ref-23] Von Ketelhodt L, Bergmann C (2010). Dual energy X-ray transmission sorting of coal. Journal of the Southern African Institute of Mining and Metallurgy.

[ref-24] Yao Y, Wu W, Yang T, Liu T, Chen W, Chen C, Li R, Zhou T, Sun C, Zhou Y, Li X (2017). Head rice rate measurement based on concave point matching. Scientific Reports.

[ref-25] Zhang Q, Zhou Y (2022). Recent advances in non-gaussian stochastic systems control theory and its applications. International Journal of Network Dynamics and Intelligence.

[ref-26] Zhao H, Shi J, Qi X, Wang X, Jia J (2017). Pyramid scene parsing network.

